# The Emerging Role of the Prokineticins and Homeobox Genes in the Vascularization of the Placenta: Physiological and Pathological Aspects

**DOI:** 10.3389/fphys.2020.591850

**Published:** 2020-11-12

**Authors:** Nadia Alfaidy, Sophie Brouillet, Gayathri Rajaraman, Bill Kalionis, Pascale Hoffmann, Tiphaine Barjat, Mohamed Benharouga, Padma Murthi

**Affiliations:** ^1^Unité 1036, Institut National de la Santé et de la Recherche Médicale, Grenoble, France; ^2^Department of Biology, University of Grenoble Alpes, Grenoble, France; ^3^Commissariat à l’Energie Atomique et aux Energies Alternatives (CEA), Biosciences and Biotechnology Institute of Grenoble, Grenoble, France; ^4^INSERM U1203, Department of Reproductive Biology, University of Montpellier, Montpellier, France; ^5^Faculty of Health and Biomedicine, First Year College, Victoria University, St. Albans, VIC, Australia; ^6^Department of Maternal-Fetal Medicine, Obstetrics and Gynaecology, Pregnancy Research Centre, Royal Women’s Hospital, The University of Melbourne, Parkville, VIC, Australia; ^7^Unité 1059, Saint-Etienne Hospital, Institut National de la Santé et de la Recherche Médicale, Saint-Étienne, France; ^8^Unité Mixte de Recherche 5249, Laboratoire de Chimie et Biologie des Métaux, Centre National de la Recherche Scientifique (CNRS), Grenoble, France; ^9^Department of Pharmacology, The Ritchie Centre, Monash Biomedicine Discovery Institute, Hudson Institute of Medical Research, Monash University, Clayton, VIC, Australia

**Keywords:** vessel development, endothelial cells, angiogenesis, prokineticins, homeobox genes, vascularization, pregnancy, EG-VEGF

## Abstract

Vasculogenesis and angiogenesis are key processes of placental development, which occur throughout pregnancy. Placental vasculogenesis occurs during the first trimester of pregnancy culminating in the formation of hemangioblasts from intra-villous stem cells. Placental angiogenesis occurs subsequently, forming new blood vessels from existing ones. Angiogenesis also takes place at the fetomaternal interface, allowing essential spiral arteriole remodeling to establish the fetomaternal circulation. Vasculogenesis and angiogenesis in animal models and in humans have been studied in a wide variety of *in vitro*, physiological and pathological conditions, with a focus on the pro- and anti-angiogenic factors that control these processes. Recent studies revealed roles for new families of proteins, including direct participants such as the prokineticin family, and regulators of these processes such as the homeobox genes. This review summarizes recent advances in understanding the molecular mechanisms of actions of these families of proteins. Over the past decade, evidence suggests increased production of placental anti-angiogenic factors, as well as angiogenic factors are associated with fetal growth restriction (FGR) and preeclampsia (PE): the most threatening pathologies of human pregnancy with systemic vascular dysfunction. This review also reports novel clinical strategies targeting members of these family of proteins to treat PE and its consequent effects on the maternal vascular system.

## Introduction

The placenta is the key organ for a successful pregnancy. It acts as a semi-permeable barrier to control nutrient and gasses exchanges and regulate waste produced by developing fetus. This hemochorial villous organ develops earlier during pregnancy, following the erosion of nearby maternal capillaries by the lytic activity of the syncytiotrophoblast. Around the 11th-12th day post-conception, the primitive uteroplacental circulation is launched (Gude et al., 2004). The establishment of this circulation is based on two key processes, the first one takes place within the placental villi and is governed by the intra-villi vasculogenesis and angiogenesis; the second one occurs at the fetomaternal interface and is governed by the extravillous trophoblasts (EVT) that remodel maternal spiral arteries to establish the fetomaternal circulation (Velicky et al., 2016).

Villous vascularization is an important process in organogenesis and is essential for the placenta to function efficiently (Zygmunt et al., 2003). At the end of the 3rd week post-conception, intra-villous stem cells differentiate into connective tissue culminating in the formation of hemangioblasts. The differentiation of the hemangioblasts into angioblasts and hematopoietic cells allows the formation of new blood vessels that connect with those of the embryo to form the primitive capillary network (Gude et al., 2004). Placental vasculogenesis is followed by two phases of angiogenesis; branching angiogenesis in immature villi where new vessels form by sprouting, and non-branching angiogenesis in the stem villi, where capillary loops form through elongation. These processes increase the surface area to volume ratio (Charnock-Jones, 2002; Chaddha et al., 2004; Zou et al., 2011) and enhance diffusional exchange between the maternal and fetal circulations (Kingdom et al., 2000; Kaufmann et al., 2004). The second type of angiogenesis, occurring at the fetomaternal interface is mainly ensured by EVTs. By the end of first trimester of pregnancy, the EVTs acquire an invasive phenotype along with markers of endothelial cells. These features allow them to colonize maternal spiral arteries, through the replacement of maternal endothelial cells, culminating in an increase in diameter of these vasculature, which allow more oxygenated maternal blood in the intervillous space (Burton and Jaunaiux, 2001; Burton, 2009).

## Impaired Placental Angiogenesis and Pregnancy-Associated Disorders

The spectrum of vascular defects associated with clinically significant pregnancy disorders including fetal growth restriction (FGR) and preeclampsia (PE), attests to the close relationship between the placental angiogenesis and embryonic development. Villi from placentae where intrauterine embryonic death and blighted ova exhibit aberrant vascular characteristics with significantly decreased vascular density, fibrosis and hydropic degeneration (Meegdes et al., 1988).

Studies using stereological techniques have reported that in placentae from FGR pregnancies, the number, surface area and volume of terminal villi were significantly reduced in FGR compared with placentae from uncomplicated pregnancies. Villous vessels exhibited fewer branches with slender and uncoiled vessels (Chen et al., 2002; Mayhew, 2003; Mayhew et al., 2003). Reduced capability of branching angiogenesis (non-branching angiogenesis) in FGR was strongly associated with a reduced supply of oxygen and nutrients to the fetus, and a subsequent delay in fetal growth (Kingdom et al., 2000; Salafia et al., 2006). Whether the vascular defects cause human FGR, or whether these changes are a consequence of aberrant biological mechanisms in the placentae from FGR pregnancies (Maulik et al., 2006) is unknown. To address this, it is vital to understand the molecular regulation of angiogenesis in the human placenta.

## Molecular Regulation of Angiogenesis

The processes of angiogenesis involve distinct changes in the phenotype of endothelial cells (ECs), which comprise the basic organizational units of vascular structures. The stimuli for these complex processes of placental angiogenesis are temporally coordinated by the microenvironment surrounding the EC surface (Patel et al., 2005). At the molecular level, *in vitro* and *in vivo* studies reported several growth factors and receptors that activate critical signaling pathways (Arderiu et al., 2007). Vascular endothelial growth factor (VEGF), placental growth factor (PlGF), and angiopoietins together with their primary receptors, VEGF receptor-1 (VEGFR-1) and VEGF receptor-2 (VEGFR-2) and PlGF that binds only to VEGFR-1, were identified as key candidates by Patel et al. (2005). VEGF is a potent stimulator of EC proliferation, migration, and production of plasminogen activators that are required for degradation of the basement membrane (Regnault et al., 2002).

In uncomplicated pregnancies, placental expression of key growth factors correlates with their established roles. For example, expression of VEGF and VEGFR-2 is highest during early gestation, which coincides with vasculogenesis and branching angiogenesis, but expression declines with advancing pregnancy (Jackson et al., 1994). Conversely, PlGF and VEGFR-1 expression is highest toward term, coinciding with non-branching angiogenesis (Clark et al., 1996).

Although differential expression of these angiogenic factors have been implicated in the development in PE and FGR (Ahmed and Perkins, 2000; Tsatsaris et al., 2003), new families of proteins have been identified and reported to play key roles in the control of these angiogenic processes. These include two important families of proteins; the prokineticins and the nuclear transcription factors including homeobox genes/homeodomain proteins.

## Prokineticin Family in the Control of Placental Angiogenesis

Recent studies from our group provided evidence for the direct role of a new placental angiogenic called prokineticin 1 (PROK1) in normal and FGR pregnancies (Hoffmann et al., 2006, 2009; Brouillet et al., 2012b, 2013b; Murthi et al., 2015; Sergent et al., 2016). Due to its similarities of action with VEGF (LeCouter et al., 2001), PROK1 is also called as endocrine gland derived-vascular endothelial growth factor (EG-VEGF). EG-VEGF belongs to the prokineticin family that includes two key members PROK1 and PROK2, also called BV8 (≈ 8 kDa) (LeCouter et al., 2001; Lin et al., 2002). These circulating ligands show differential expression patterns in humans depending on the organ/tissue types. For example, PROK1/EG-VEGF is highly expressed in peripheral tissues (specifically in steroidogenic organs such as human ovary, placenta and adrenal gland), whereas PROK2/Bv8 is widely expressed in the central nervous system and non-steroidogenic cells of the testes (LeCouter et al., 2001; Lin et al., 2002; Traboulsi et al., 2015). EG-VEGF and BV8 activate two G-protein linked receptors namely prokineticin receptor 1 (PROKR1) and -2 (PROKR2). The signaling pathways include, cAMP, Akt, and p42-p44 MAP-kinases phosphorylation and calcium mobilization. PROKs regulate a stunning array of biological functions such as gastrointestinal motility (LeCouter et al., 2001; Lin et al., 2002), circadian rhythm regulation, neurogenesis, angiogenesis, pain perception, mood regulation, and reproduction (Brouillet et al., 2010, 2012b; Alfaidy et al., 2016; Zhao et al., 2019). Dysregulation of PROKs/PROKRs signaling pathways have been reported in a variety of diseases, such as cancer, abnormal angiogenesis and pregnancy pathologies (Brouillet et al., 2010, 2012b; Alfaidy et al., 2016; Traboulsi et al., 2017; Zhao et al., 2019).

Throughout normal human pregnancy, a dynamic expression of circulating EG-VEGF is found in the serum of pregnant women, with a five-fold increase during the first trimester (≈250 pg/ml) (Hoffmann et al., 2009). The placental expression of EG-VEGF is high during the first trimester of human pregnancy, with a peak at 8–11 weeks of gestation (Hoffmann et al., 2009). PROKR1 and PROKR2 are highly expressed in villous cytotrophoblasts (VCT) as well as micro- and macrovascular placental endothelial cells (Brouillet et al., 2010). At the cellular level, EG-VEGF is expressed in the syncytiotrophoblast (ST), VCT, fetal endothelium, and Hofbauer cells (Ho) (Hoffmann et al., 2006; Holloway et al., 2014; Garnier et al., 2015).

## Prokineticins Expression in Micro- and Macrovascular Placental ECs

**FIGURE 1:**
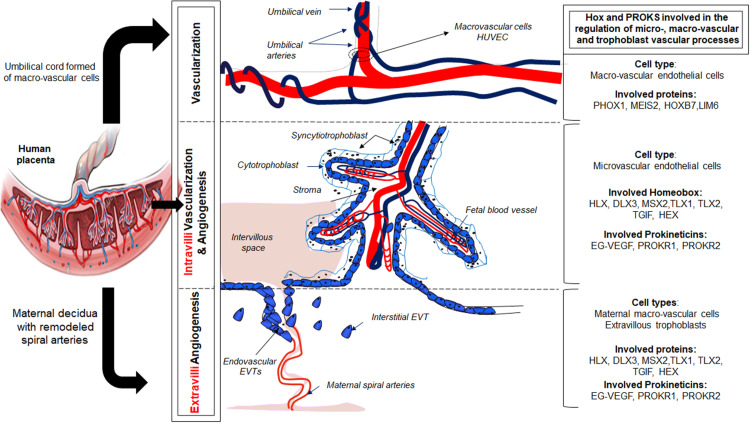
Illustration of the role of the prokineticin and HOX family members in the control of placental vascularization and angiogenesis.

Lang et al. (2003) reported distinct morphogenetic, antigenic and functional differences between the two EC types present in the placenta, with respect to the secretion of vasoactive substances and the proliferative response to cytokines. The dissimilar responses of micro- and macrovascular ECs to various stimuli (Lang et al., 2003; Brouillet et al., 2010) most likely reflect differences in the activation of transcription factors, Figure 1. Despite these differences, HUVEC are the predominant cell type used to model placental vasculogenic and angiogenic processes (Demir et al., 1989). Microvascular ECs, despite being the predominant cell type that vascularize the placental villi are less well understood. Importantly, these cells play critical role in placental disorders such as FGR and PE (Demir et al., 1989; Kingdom et al., 2000; Lang et al., 2003).

Endocrine gland derived-vascular endothelial growth factor enhances angiogenesis within the placental villi during the first trimester of human pregnancy (Brouillet et al., 2010). It controls various angiogenic processes including endothelial cell proliferation, survival, migration, tube organization, sprouting, permeability, and paracellular transport (Brouillet et al., 2010). Interestingly, EG-VEGF also displays stronger effects on the placental microvascular endothelial cells, the PLEC cells compare to its effects on the macrovascular cells, the HUVEC (Human umbilical vessel endothelial cells) (Brouillet et al., 2010). Via its PROKR1 receptor, EG-VEGF increases PLEC proliferation, migration and sprouting and controls their permeability via PROKR2. Importantly, EG-VEGF effects on PLEC cells were stronger compare to the VEGF effects on the same cells (Brouillet et al., 2010). At the feto-maternal interface, we demonstrated that EG-VEGF controls extra-villi angiogenesis through the inhibition of precocious invasion of EVT into the maternal spiral arteries (Hoffmann et al., 2009).

Recent studies from our own group and from others demonstrated that increased expression of EG-VEGF is associated with PE and FGR development (Hoffmann et al., 2006; Alfaidy, 2016; Inan et al., 2018). Our group also demonstrated that the concentration of EG-VEGF is significantly increased in both pathologies (Hoffmann et al., 2006; Murthi et al., 2015; Alfaidy, 2016). More recently, a study confirmed the increase in EG-VEGF levels in PE and proposed this factor as a biomarker for the diagnosis of PE patients (Inan et al., 2018).

## Key Regulators of Prokineticins for Successful Placental Angiogenesis

Since its identification EG-VEGF has been associated with the control of placental angiogenesis during the first trimester of pregnancy and the reactivation of its receptors in the placenta of patients with pregnancy pathologies such as PE and FGR (Brouillet et al., 2010, 2012a, 2013a, 2014a,b; Garnier et al., 2015; Alfaidy, 2016; Sergent et al., 2016). The reactivation of angiogenic processes was meant to compensate for the associated deleterious vascularization. In these pathologies, EG-VEGF and receptors contribute to neoangiogenesis processes that allow pregnancy to progress. To fulfill these functions, EG-VEGF and its receptors have been reported to be regulated by key actors of vasculogenesis and angiogenesis within the placenta. The first supposed regulator of EG-VEGF expression was oxygen. This was reported by Ferrara et al. (LeCouter et al., 2001), as HIF1α response element was identified in the promoter region of EG-VEGF and BV8. In 2006, we demonstrated that EG-VEGF and its PROKR1 receptor were upregulated by hypoxia in the human placenta (Hoffmann et al., 2006). EG-VEGF upregulation by hypoxia substantiated its role during the first trimester, as placental development and vascularization occur in hypoxic environment during this period (Burton, 2009). In 2012, we demonstrated that another key actor of placental development, the human chorionic gonadotropin (βhCG) upregulates the expression of EG-VEGF and its receptors (Brouillet et al., 2012b). Importantly, in 2013, glycosylated-hCG has been reported to regulate another actor of angiogenesis, the TGFb (Berndt et al., 2013). These finding suggest that EG-VEGF belongs to a complex of placenta proteins that are controlled by the master hormone, βhCG to fulfill a well orchestrated angiogenesis. In the same lane, we have also demonstrated in 2015 that part of the effects of the transcription factor PPARγ on placental angiogenesis are mediated by EG-VEGF, suggesting that the placental defects observed in the PPARγ knockout mice might well be due to deregulations in the EG-VEGF/PROKR functions (Garnier et al., 2015).

## Transcriptional Factors in the Control of Placental Angiogenesis

Cells respond to cues from growth factors and signaling molecules, allowing them to either maintain or alter their state of differentiation during angiogenesis (Irving and Lala, 1995). However, EC nuclear transcription factors determine how these cues are interpreted and drive the cellular response. Many different types of transcription factors play essential roles in placental cell differentiation, including endothelial and trophoblast cells (Cross et al., 2002). Most transcription factors have common protein structural motifs allowing them to be placed into a few large families (e.g., zinc finger, leucine zipper, helix-loop-helix and helix-turn-helix) (Johnson and McKnight, 1989; Woodside et al., 2004). This review will mainly focus on members of the “homeobox” gene family of transcription factors.

## Homeobox Genes and Homeodomain Proteins

Homeobox genes are frequently present as clusters of related homeobox genes called “HOX” cluster genes, but there are also individual, divergent HOX-like genes. Homeobox genes contain a highly conserved 180 base pair DNA sequence, which encodes a 60 amino acid “homeodomain” and contains a helix-turn-helix DNA binding motif. Although homeodomain proteins have similar DNA binding specificity, they regulate highly diverse and context-dependent cellular functions (Levine and Hoey, 1988), which includes the processes of vasculogenesis and angiogenesis.

## Homeobox Genes in Murine and Human Placental Development

Targeted deletion of specific homeobox genes in murine models provides genetic proof of homeobox gene regulation of placental development during pregnancy (Rossant and Cross, 2001; Myers and Capper, 2002; Gorski and Leal, 2003; Gorski and Walsh, 2003). For example, targeted deletion of Esx1 (Fohn and Behringer, 2001) and Dlx3 (Morasso et al., 1999) resulted in disruption of the vascular network in the placental labyrinthine layer (Cross et al., 2003). Mutant embryos in both cases were growth-restricted, and Dlx3-/- mutants were embryonic lethal due to adequate placental circulation (Morasso et al., 1999). These studies show homeobox genes are specific regulators of placental vascular development.

Recent studies from our laboratory provided comprehensive analyses of homeobox genes in human placental pathologies. We carried out the first screening of a 32-weeks placental cDNA library for homeobox genes, which led to the isolation of DLX4, MSX2, GAX and HLX (Quinn et al., 1997). Immunohistochemical analyses localized these homeobox genes/homeodomain proteins to trophoblasts and ECs (Murthi et al., 2006; Rajaraman et al., 2008; Chui et al., 2010). We also reported decreased homeobox gene HLX expression in ECs and trophoblast cells in FGR-affected placentae (Murthi et al., 2006). Homeobox genes DLX4 and DLX3 showed increased expression in FGR-affected placentae (Murthi et al., 2006), whereas GAX and MSX2 showed no significant difference in expression.

## Homeobox Gene Expression in Micro- and Macrovascular Placental ECs

In our studies, primary PLEC were used to identify homeobox genes expressed in the placental microvasculature, and to compare that with macrovascular HUVEC. We detected mRNA expression of homeobox genes HLX, MSX2, DLX3, DLX4, and GAX and in both PLEC and HUVEC. Notably, HLX mRNA in HUVEC was significantly lower compared with PLEC (Murthi et al., 2007). These data provided evidence of heterogeneity in homeobox gene expression between microvascular PLEC and macrovascular HUVEC, which most likely reflects significant differences in EC function in the two different cellular environments, Table 1.

**TABLE 1:** Summarizes the localizations of the prokineticin and HOX members within the placenta, their respective roles in the control of the angiogenesis in macro and microvascular blood vessels and lists their local regulators.

Placental prokineticins and homeobox genes	Role in the placenta	Type of placenta associated structure	Regulated genes in the placenta		Placental regulators	Associated placental vascular pathologies	References
EG-VEGF, PROKR1 and PROKR2	Angiogenesis, inflammation Placental development	Trophoblast cells, micro and macrovascular systems, Hofbauer cells	HOXD1, 8, 9, and 11 HOX A9 and HOXC8, 10		Nicotine, Hypoxia, βhCG, PPARg	FGR, Hydatidiform moles, Choriocarcinoma and PE	Hoffmann et al., 2006, 2009; Brouillet et al., 2012a, b, 2013b; Holloway et al., 2014; Garnier et al., 2015; Murthi et al., 2015; Traboulsi et al., 2015, 2017; Sergent et al., 2016
HLX, DLX3, DLX4, MSX2, GAX, TLX1, TLX2	Cell Invasion, migration andproliferation Stem cell proliferation and differentiation	Microvascular system	CDKN1C (+) RB (+) GATA2, PPAR g ITGAV, NRP-1, ANPGT-1 and 2	VEGF, PLGF, HGF, CSF-1 Angiopoietins, PPARg, IGF-II, Endoglin, TGFb	IGFR2 (-) PLGF (+)	FGR and PE	Murthi et al., 2007, 2008; Rajaraman et al., 2008, 2010; Chui et al., 2013; Liang et al., 2016; Novakovic et al., 2017; Harris et al., 2019
TGIF		Micro and macrovascular systems				FGR (+)	Pathirage et al., 2013; Gunatillak et al., 2016
HEX	Role in hematopoiesis	Vascular system					Unpublished data
PHOX1		Macrovascular system					Unpublished data
MEIS2	Control of mouse placental vascularization	Macrovascular system	Activin and Inhibin				Unpublished data
LIM6		Macrovascular system	VEGF				Unpublished data
HOXB7	Endothelial differentiation	Macrovascular system			DKK1 (−) Wnt1/b catenin (−)	FGR (+)	Huang et al., 2019
NKX3.1	Role in trophoblast differentiation and proliferation	Trophoblast lineage			EG-VEGF (−)		Murthi et al., 2015
HOXD1, 8, 9, and 11 HOX A9 and HOXC8, 10			HOXA9 upregulates MMP14, EphB4, eNOS, VEGFR2		EG-VEGF (+)	FGR (+)	Murthi et al., 2015

*Also reports their associated placental vascular pathologies.*

## Functional Significance of Homeobox Genes in Micro- and Macrovascular ECs

We detected high HLX mRNA expression in PLEC, which are also proliferative cell types compared with their macrovascular counterparts (Murthi et al., 2007). Moreover, in response to placental growth factor (PlGF), PLEC have more proliferative activity compared with HUVEC (Lang et al., 2003; Brouillet et al., 2010). Together, these data suggest a possible role for HLX in the proliferative capacity of microvascular ECs. The role(s), if any, of homeobox genes HLX, MSX2, DLX3, DLX4, and GAX in the transcriptional regulation of other PLEC function such as migration and invasion is yet to be explored. Quinn et al. (2000) proposed that co-expression of a combination of homeobox genes (i.e., HLX, MSX2, and GAX) may play a role in the regulation of epithelial-mesenchymal interactions in the placenta. Thus, co-expression of the homeobox genes in both trophoblast and endothelium may also be important in coordinating villous outgrowth and angiogenesis within the terminal villi. Other studies showed that Homeobox genes regulate numerous key genes such as, CDKN1, RB, GATA2, PPARg, ITGAV, NRP-1, ANGPT-1, and 2 (Rajaraman et al., 2008; Chui et al., 2013; Novakovic et al., 2017; Harris et al., 2019; Table 1).

The repertoire of homeobox genes expressed in PLEC, was further investigated by microarray expression profiling of ECs (Murthi et al., 2008). We identified homeobox genes TLX1, TLX2, TGIF, HEX, PHOX1, MEIS2, HOXB7, and LIM6 in PLEC. Importantly, our studies reported that these homeobox genes were differentially expressed in macro- compared with microvascular ECs, Figure 1. Functional studies in cultured ECs are underway in our laboratory to determine the role of these novel endothelial homeobox genes.

## Growth Factor Regulation of Homeobox Genes in the Placenta

Many EC growth factors and signal transduction pathways are involved in the maintenance of an efficient uteroplacental vasculature (Thaete et al., 2004). Our previous studies have demonstrated that HLX expression in *in vitro* models of human EVT, HTR8-SV neo and SGH-PL4 was significantly upregulated by HGF (Rajaraman et al., 2010) and CSF-1 (Rajaraman et al., 2007, 2008). Recent studies also showed that the homeobox gene HOXB7 is regulated by DKK1 and the Wnt-1/b-catenin (Huang et al., 2019; Table 1).

In 2015, our group demonstrated that the canonical member of the prokineticin family, EG-VEGF, controls homeobox genes expression in normal human placenta and in placenta from FGR pregnancies (Murthi et al., 2015). This regulation was observed in whole placenta explants, including endothelial, stroma and trophoblast cells. In particular, EG-VEGF up-regulated the following homeobox genes, HOXA9, HOXC8, HOXC10, HOXD1, HOXD8, HOXD9, and HOXD11, while downregulating the expression of NKX 3.1. Further investigations using an *in vitro* model of trophoblast cells, we demonstrated that reduced NKX3.1 expression significantly enhanced premature differentiation and apoptosis in the syncytiotrophoblast cell line, the BeWo, while significantly reduced migration and invasive potential of the HTR8-SV neo cells (Murthi et al., 2015). This study was the first to demonstrate that the new placental angiogenic factor exhibits part of its effects on trophoblast invasion and differentiation through the NKX3.1 homeobox gene. The demonstration that growth factors such as EG-VEGF regulate homeobox genes in the trophoblast lineage, especially the EVT, involved in extravillous angiogenesis, opens new perspectives into the potential involvement of homeobox genes in the cross talk between trophoblast and extra-villi and intra-villi endothelial cells to fulfill placental angiogenesis throughout pregnancy. Ongoing validations of the newly discovered EG-VEGF-regulated homeobox genes should bring more insights into the role of these homeobox genes in the control of angiogenic processes at both intra-villi and extra-villi sites, throughout human pregnancy, Figure 1.

## Prokineticins and/or Homeobox Genes as Potential Targets in FGR and PE Pathologies

Numerous studies from our team have clearly demonstrated that EG-VEGF and its receptors are directly involved in normal placental vascularization and angiogenesis during the first trimester of pregnancy and that maintenance of increased circulating levels of placental EG-VEGF over that trimester is associated with the development of PE and FGR (Hoffmann et al., 2006, 2009; Brouillet et al., 2012b; Murthi et al., 2015; Traboulsi et al., 2015). These finding strongly suggest that antagonisation of EG-VEGF signaling might contribute to the attenuation of vascular-pregnancy pathologies. Importantly, we have recently demonstrated that treatment of animal model of choriocarcinoma with PROKR2 antagonist significantly reduced tumor growth, vascularization and metastasis (Traboulsi et al., 2017). Hence, one can speculate that the benefit upon the antagonisation of the prokineticin signaling might well trigger an upstream regulation of the EG-VEGF-dependent homeobox genes including NKX3.1, HOXA9, HOXC8, HOXC10, HOXD1, HOXD8, HOXD9, and HOXD11 as potential target genes for aberrant angiogenesis associated with the pathogenesis of PE and FGR. Further studies are needed to better characterize the relationship between placental angiogenic factors and the homeobox genes to fulfill successful pregnancy outcomes.

## Author Contributions

NA and PM designed the manuscript, supervised the progress of the review, and verified the English aspects. SB, GR, and BK wrote the different parts of the review. MB, TB, and PH helped with the clinical aspects and performed the figure and table. All authors contributed to the article and approved the submitted version.

## Conflict of Interest

The authors declare that the research was conducted in the absence of any commercial or financial relationships that could be construed as a potential conflict of interest.
